# Mucin promotes Psl production and surface exploration in *Pseudomonas aeruginosa*

**DOI:** 10.1128/mbio.01445-26

**Published:** 2026-08-13

**Authors:** Sabrina I. Lamont, Matthew J. Pestrak, Matthew R. Parsek, Katharina Ribbeck, Daniel J. Wozniak

**Affiliations:** 1 Department of Microbiology, Ohio State University 2647 , Columbus, Ohio, USA; 2 Department of Microbial Infection and Immunity, Ohio State University College of Medicine 12305 , Columbus, Ohio, USA; 3 Department of Microbiology, University of Washington School of Medicine 12353, Seattle, Washington, USA; 4 Department of Biological Engineering, Massachusetts Institute of Technology 2167, Cambridge, Massachusetts, USA; Universite de Geneve, Geneva, Switzerland

**Keywords:** Psl, surface exploration, mucin, biofilms, twitching

## Abstract

In chronic, recalcitrant infections, *Pseudomonas aeruginosa* forms biofilm aggregates that are encapsulated in a protective extracellular matrix made of exopolysaccharides, extracellular DNA, and matrix proteins. Biofilm formation is a major cause of antibiotic clearance failure in *P. aeruginosa*, and there is growing pressure to elucidate biofilm preventative measures and develop novel anti-biofilm treatments. Toward this goal, much work has been done to understand the genetic adaptations *P. aeruginosa* undergoes in chronic respiratory infections, but how the mucosal environment, found in the lung, impacts *P. aeruginosa* behavior is unclear. Mucus is a protective hydrogel lining wet epithelial cells and is mainly composed of water and its structural component, the glycoprotein mucin. Using commercially purified porcine gastric mucin, which is missing its end tail regions and has modified glycosylation, as a model of structurally altered mucus, we show that *P. aeruginosa* rapidly increases surface exploration via twitching motility, depositing trails of the exopolysaccharide Psl and elevating total Psl levels. This increased Psl production occurs on short timescales (<1 h), is independent of transcriptional or translational regulation, and enhances tolerance to select antimicrobials, including hydrogen peroxide and ciprofloxacin. Together, these findings indicate that structurally altered mucus environments function as contextual signals that elicit rapid, non-genetic bacterial adaptive behaviors relevant to persistence in chronic infection.

**IMPORTANCE:**

Understanding how the host environment impacts pathogen behavior is crucial for developing effective treatments for infections. Here, we provide evidence that the mucosal glycoprotein, mucin, and the O-glycans that decorate the mucin protein backbone alter surface exploration by stimulating twitching motility in the opportunistic pathogen *Pseudomonas aeruginosa*. This increase in twitching then results in an extensive network of Psl trails, which are a precursor to microcolony formation. Mucin additionally promotes Psl production in a novel post-translational manner across a small cohort of isolates, suggesting Psl production by mucin is conserved. This increase in Psl production was found to also protect cells from killing by antimicrobials. Independent of mucin, we present evidence that the secondary messenger, c-di-GMP, can also post-translationally regulate Psl production, which is the first evidence of post-translational regulation for this exopolysaccharide.

## INTRODUCTION

**Fig 1:**
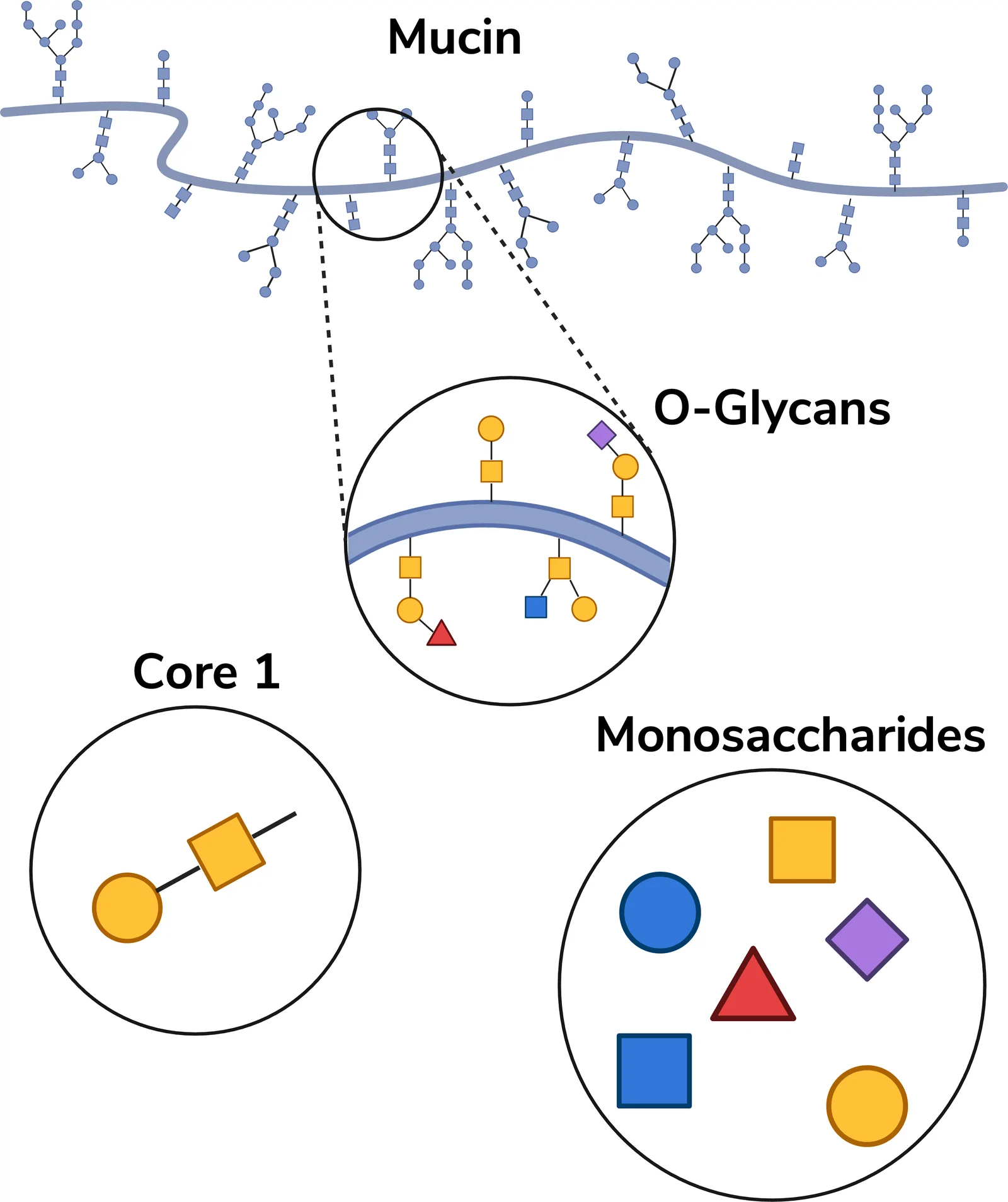
Mucin and its components: representative diagram portraying the glycoprotein mucin decorated with O-glycans of various structures, lengths, monosaccharides, and modifications. One of the most common glycan structures found in mucin is the core 1 structure represented by a yellow circle (Gal) and yellow square (GalNAc). These glycans consist of the monosaccharides galactose (yellow circle), N-acetyl-galactosamine (yellow square), N-acetyl-glucosamine (blue square), mannose (green circle), fucose (red triangle), and sialic acid (purple diamond).

*Pseudomonas aeruginosa* is a pathogen proficient in colonizing a wide variety of sites throughout the human body, including respiratory and urinary tracts, bloodstream, ocular regions, and even wounds (1–4). Many of these infection sites are lined with a layer of mucus, a protective hydrogel found on every wet epithelial surface in the body. Mucus is mainly composed of water, with additional glycoproteins, antimicrobial proteins, and salts (5). Despite its significance, the impact of mucus on *P. aeruginosa* physiology and behavior is largely unexplored. Recent work has demonstrated that mucus is more than just a physicochemical barrier to potentially harmful microbes but can also serve as an environmental signal that can attenuate virulence (6–10). It is specifically the glycoprotein mucin (MUC), which allows for mucosal hydrogel structure, that serves as the main signal for altering pathogenic behavior (6–8, 11). Mucin is a large glycoprotein consisting of a proteinaceous backbone that is heavily decorated with O-glycans (Fig. 1). Mucin structure and glycosylation are altered with disease, with mucins from cystic fibrosis (CF) sputum containing higher amounts of proteolytic cleavage and increased levels of sialylated and sulfated glycans (12). While intact, natively secreted mucins suppress virulence behaviors in multiple pathogens, commercially purified mucins differ substantially in polymer architecture, hydrogel formation, and glycosylation, including increased sialylated glycans due to industrial processing (13–15). These materials therefore model structurally altered or compromised mucus states rather than healthy mucus barriers. Because mucus integrity is frequently disrupted during chronic infection and inflammation, understanding how *P. aeruginosa* responds to altered mucin is critical for interpreting bacterial behavior in disease-relevant contexts (5, 13, 16, 17).

A key feature of chronic virulence in *P. aeruginosa* is biofilm formation; a multicellular community encapsulated in a protective extracellular matrix. In *P. aeruginosa*, this matrix is mainly composed of exopolysaccharides (Pel, Psl, and/or alginate), extracellular DNA, and matrix proteins like CdrA (18). The exopolysaccharide Psl is a pentasaccharide composed of L-rhamnose, D-mannose, and D-glucose. It is important for surface adherence, biofilm maintenance, and can confer protection against antimicrobials and host defenses (19–25). CF sputum samples regularly contain Psl, and it is produced by clinical isolates from various mucosal sites (26, 27). Additionally, Psl expression is associated with worsened outcomes in pediatric patients with CF (28). This highlights the importance of Psl in chronic infections and indicates that Psl is expressed and synthesized in a mucosal environment. Previously, mucin was shown to reduce *P. aeruginosa* biofilm formation on abiotic surfaces and adhesion to A549 epithelial cells (29, 30). Bacterial surface contact is a strong signal for biofilm initiation and subsequent EPS production, but the influence of mucin on this process is currently unexplored.

In this study, we examine how exposure to structurally altered mucin environments influences early surface exploration and matrix production by *P. aeruginosa* at the single-cell level. We found that mucin stimulated Psl production in a post-translational regulatory manner, suggesting the potential for a rapid cellular response to the host environment. Elevated Psl production is usually associated with sessility and increased biofilm production. Thus, we were surprised to find that mucin also rapidly stimulates cells to induce surface-dependent twitching motility. These twitching cells deposited a robust “trail” of Psl on such surfaces. These Psl trails have been previously suggested to facilitate twitching motility-mediated cellular aggregation on a surface (31). Besides being deposited by twitching cells, elevated Psl in mucin also appears to contribute to antimicrobial protection, specifically against H_2_O_2_ and ciprofloxacin. Collectively, these data indicate that *P. aeruginosa* possesses a rapid mechanism to induce Psl production and protect single cells in the adverse host environment. These findings suggest that compromised mucus environments can rapidly reshape bacterial behavior in ways that promote persistence and antimicrobial tolerance during infection.

## RESULTS

### Mucin induces Psl trails

**Fig 2:**
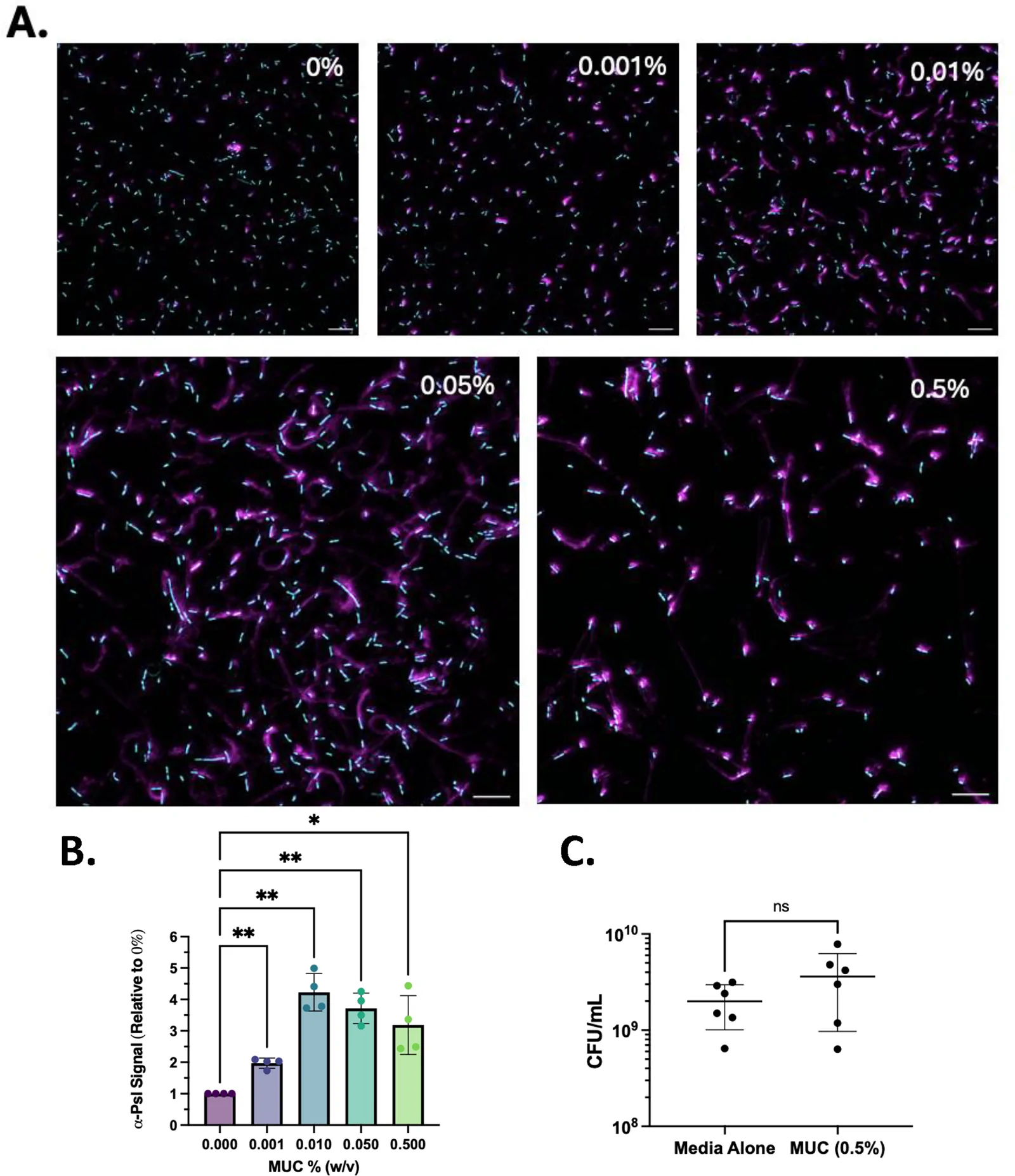
Mucin stimulates Psl trails. (**A**) Representative images of PAO1 cells that were exposed to a surface in the presence or absence of porcine gastric mucin (MUC) for 1 h at increasing concentrations. Cells were then stained with ɑ-Psl antibodies and imaged via widefield fluorescent microscopy. Distinct Psl trails are identified with arrows. Scale bar = 20 μm. (**B**) Total ɑ-Psl signal was quantified per image in FIJI and made relative to media alone (0%). *N* = 4 and *n* = 3. Significance determined via RM one-way analysis of variance with Geisser-Greenhouse correction and Tukey’s multiple comparison test. **, *P* < 0.01; *, *P* < 0.05; and ns, not significant. (**C**) Enumeration of cells exposed to mucin or media for 1 h. *N* = 2 and *n* = 6. ns determined via unpaired *t*-test *P* < 0.05.

**Fig 3:**
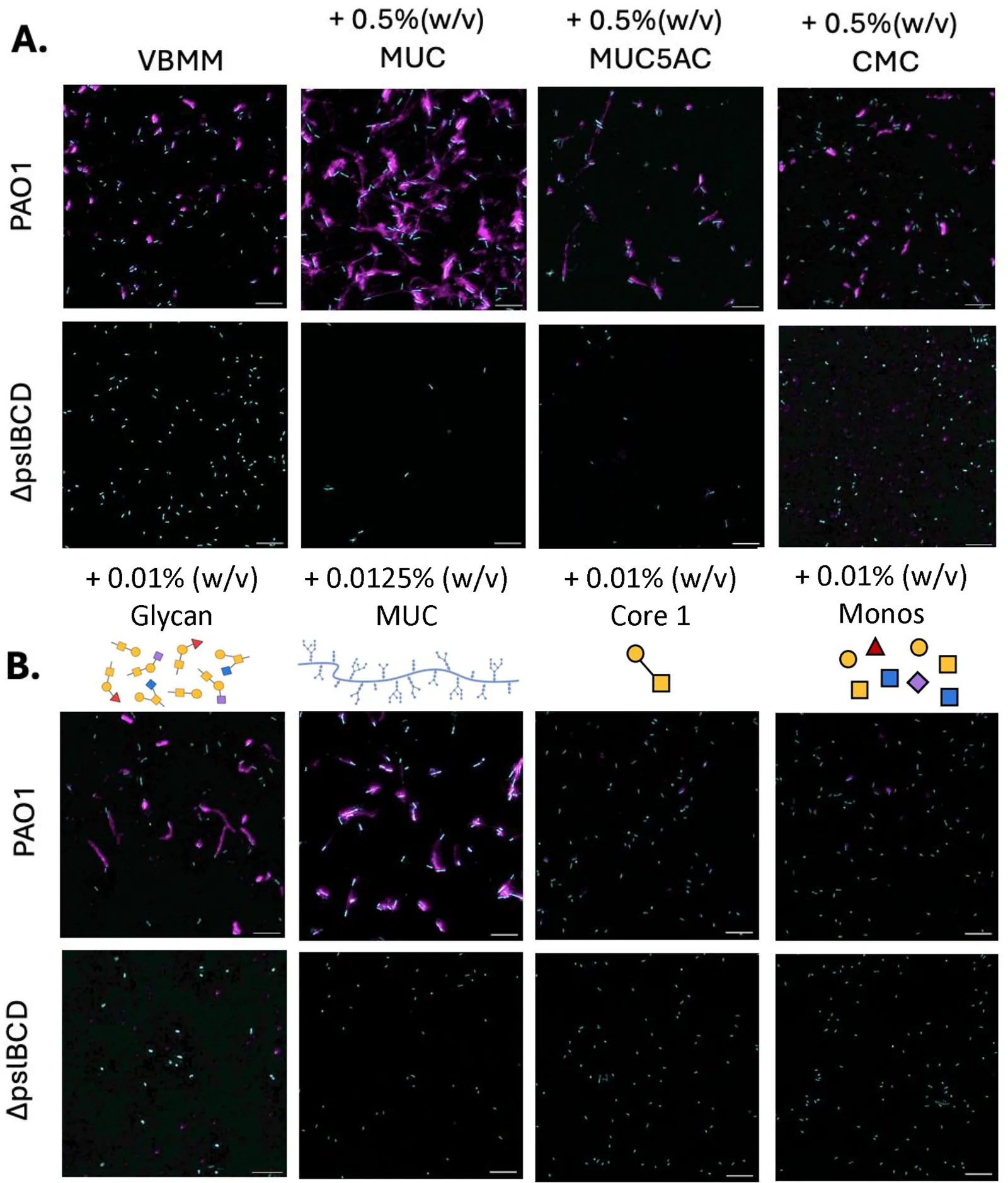
Mucin-glycans are capable of stimulating Psl trails. (**A**) Representative images of PAO1 or Δ*pslBCD* (cyan) exposed to a surface in Volger-B minimal media (VBMM), 0.5% (wt/vol) MUC, 0.5% (wt/vol) MUC5AC, or 0.5% (wt/vol) carboxymethylcellulose (CMC) before staining with α-Psl (magenta) and imaging Psl-trails on widefield fluorescent microscopy (60×). Scale = 10 μm. (**B**) Psl trails were examined at an equivalent mucin concentration to that of tested glycans, 0.01% core 1 glycans, or 0.01% (wt/vol) monosaccharides (monos).

Psl exists in a cell-associated form that is crucial for adhesion to both abiotic and biotic surfaces (22, 32). Additionally, as a cell travels across a surface, deposits of Psl, known as Psl trails (31), are left behind. These trails play an important role in surface multi-cellular organization and microcolony formation, which are necessary steps leading to robust biofilm formation (31). Both natively purified and commercially prepared mucin can interfere with surface attachment and subsequent biofilm formation, but the underlying mechanism is not fully understood (8, 33). To further investigate this, we examined *P. aeruginosa* single-cell surface behavior in mucin early after attachment (~1 h). Mucin promoted Psl trail formation in a concentration-dependent manner (Fig. 2A). As mucin concentrations increased, the morphology of Psl trails changed. At lower mucin concentrations, trails exhibited a diffuse pattern, while at higher mucin concentrations, they exhibited a tight strand-like appearance (Fig. 2A). Except for the highest concentration (0.5% [wt/vol]), increasing mucin concentrations correlated with an increase in Psl signal (Fig. 2B). The increase in surface Psl signal was not due to increased growth during the 1 h exposure, as bacterial numbers were comparable between the untreated and mucin-treated group when planktonic cells were exposed to mucin, which has also been seen previously by other groups (29, 30) (Fig. 2C). Additionally, we found that the natively purified respiratory and gastric mucin, MUC5AC, also promoted Psl trail formation, although the degree of Psl staining is less pronounced in comparison to the commercially purified porcine gastric mucin (Fig. 3A). The reduced magnitude of Psl trail formation observed with natively purified MUC5AC vs commercially purified MUC is consistent with known differences in polymer integrity and glycosylation, supporting the idea that bacterial responses are sensitive to mucus state rather than mucin presence alone (14, 34). We hypothesized that a mucin-dependent increase in viscosity at the surface could be the signal inducing Psl production and trail formation. However, a viscosity mimic of mucin, carboxymethylcellulose (CMC), did not promote trail formation or result in increased Psl signal (Fig. 3A; Fig. S2).

### Mucin-glycans induce Psl trails

Mucin is mainly composed of a long proteinaceous backbone that is decorated with O-linked glycans. These glycans vary in composition, length, higher-order structure, and modifications, and ultimately make up around 80% of the mucin molecular weight. Since pathogenic traits attenuated by mucin, such as surface adhesion and biofilm formation, have been attributed to these O-glycans, we hypothesized that mucin glycans present in partially degraded mucins found in commercial preparations were involved in stimulating Psl trail formation (14). To test this, O-glycans were cleaved via non-reductive alkaline beta-elimination ammoniolysis from the mucin backbone of commercial preparations. This allowed us to generate a pool of glycans that retains structural complexity and discern glycan-associated cues independent of the polymer and any hydrogel-driven effects. We observed that these glycans also induced Psl trails to a similar degree as a comparable concentration of commercial mucin (0.0125% [wt/vol]) and that this was also independent of growth (8, 35) (Fig. 3; Fig. S3 and S7). We next tested one of the most common glycan linkages present on mucin, the core 1 structure, but observed no Psl trail formation, suggesting that a subset of higher-order glycan features or another specific glycan structure is necessary to provide the appropriate contextual cue. Finally, only mucin-glycans, but not a pool of their constituent monosaccharides, elicited Psl-trails, suggesting glycan-associated features, rather than the sugar composition alone, are required for this response. Such features could potentially include altered sulfation patterns, changes in sialylation state, or heterogeneous glycan lengths that become more prominent when mucins are structurally compromised. Taken together, the ability of both partially degraded commercial mucin and its released O-glycan pool to elicit Psl trails indicates that the responsible cue is glycan associated and does not require an intact mucin polymer. The absence of an effect with monosaccharides or core 1 suggests that this cue likely arises from a subset of higher-order glycan structures or from ensemble properties that become more prominent when mucins are structurally compromised.

### Mucin-stimulated Psl trails are dependent on twitching motility

**Fig 4:**
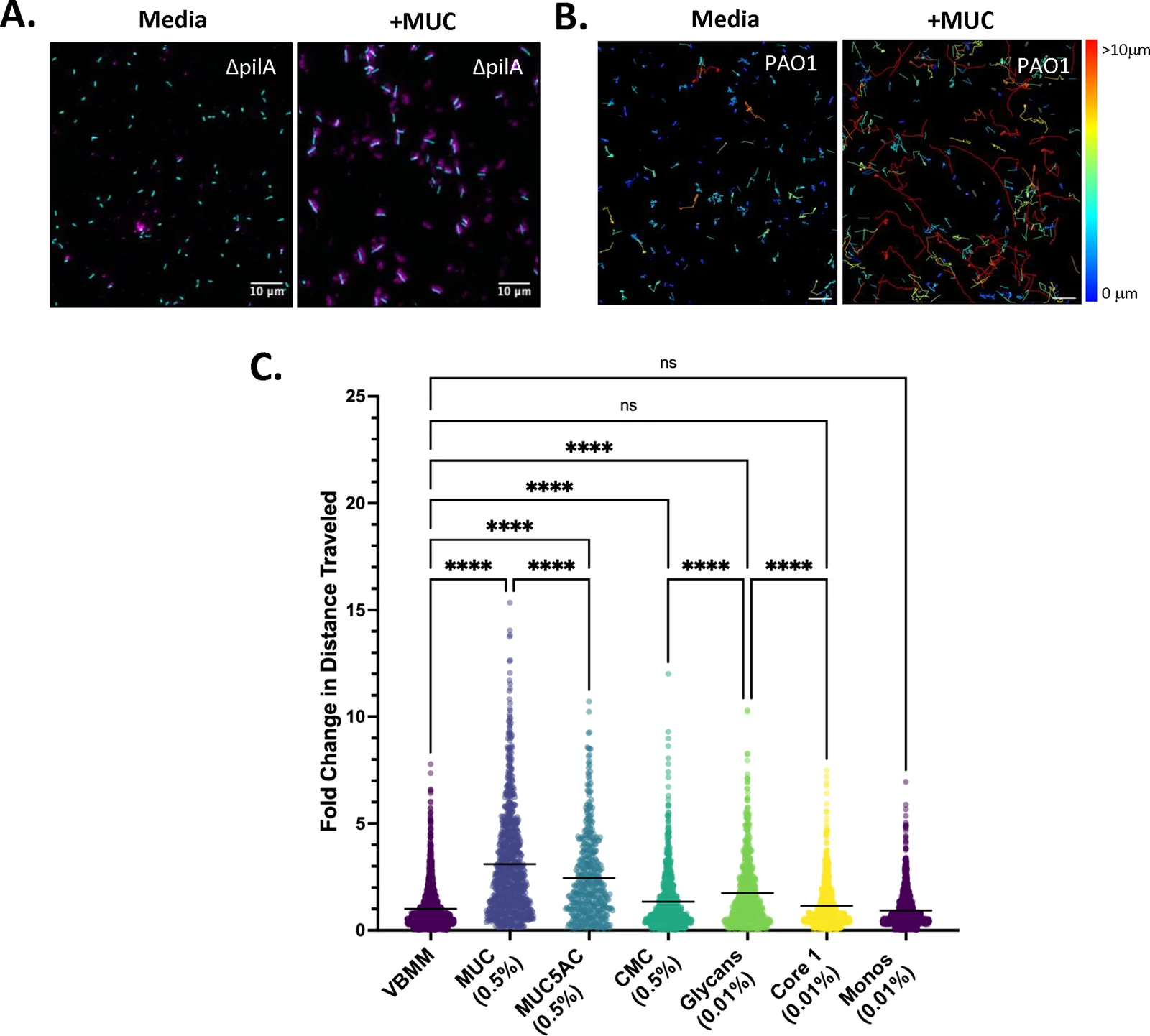
Psl trails are dependent on twitching motility. (**A**) Representative ɑ-Psl staining (magenta) of ΔpilA post 1 h exposure to 0.5% (wt/vol) mucin (MUC). Images taken via widefield microscopy at 60×. Scale bar = 10 μm. (**B**) Reconstructed trajectories of PAO1 traveling across a surface in Volger-B minimal media (VBMM) ± 0.5% (wt/vol) MUC. Cells are color-coded by distance traveled (blue, 0 μm; red, >10 μm). Scale bar = 10 μm. (**C**) Fold change in distances traveled by PAO1 in MUC, MUC5AC, carboxymethylcellulose (CMC), mucin glycans (glycans), core 1 glycan structure, and a pool of monosaccharides relative to media alone. *n* = 3. Significance was determined via ordinary one-way analysis of variance with Tukey’s multiple comparisons test, ****, P < 0.0001; ns, not significant.

Previous studies demonstrated that Psl trails occur as a cell deposits Psl onto a surface while employing twitching motility to move (31). To determine whether the Psl surface deposits we observed in the presence of mucin result from a similar mechanism, a twitching motility-deficient mutant (Δ*pilA*) was exposed to 0.5% (wt/vol) mucin and stained for Psl (Fig. 4A). No Psl trails were observed, though increased Psl staining was observed in mucin, indicating that type IV pili are also necessary for Psl trail formation in mucin. This led to the question of whether mucin could be promoting twitching motility itself. To test this, cell tracking of surface-associated cells was performed every 2.5 min over the course of 1 h in the presence or absence of mucin (Fig. 4B; Fig. S4). Compared to media alone, we observed that more cells twitch and travel greater distances in mucin (Fig. 4C; Video S1). A Δ*pslBCD* mutant also twitches further in mucin, though not to the same extent as the isogenic wild type (Fig. S4 and S9). The viscosity mimic, CMC, was also able to induce a statistically significant increase in distance traveled (1.34-fold increase), though not to the same degree as mucin-glycans (1.74-fold increase), suggesting viscosity could partially contribute to mucin-dependent increases in twitching motility. Consistent with the Psl staining results, the core 1 glycan structure was insufficient to induce twitching under the conditions tested. We also confirmed that increased twitching distances stimulated by glycans were not due to the individual sugar components, since a pool of monosaccharides also did not promote mucin-dependent increases in twitching motility (Fig. 4C).

### Mucin stimulates Psl production at the post-translational level

**Fig 5:**
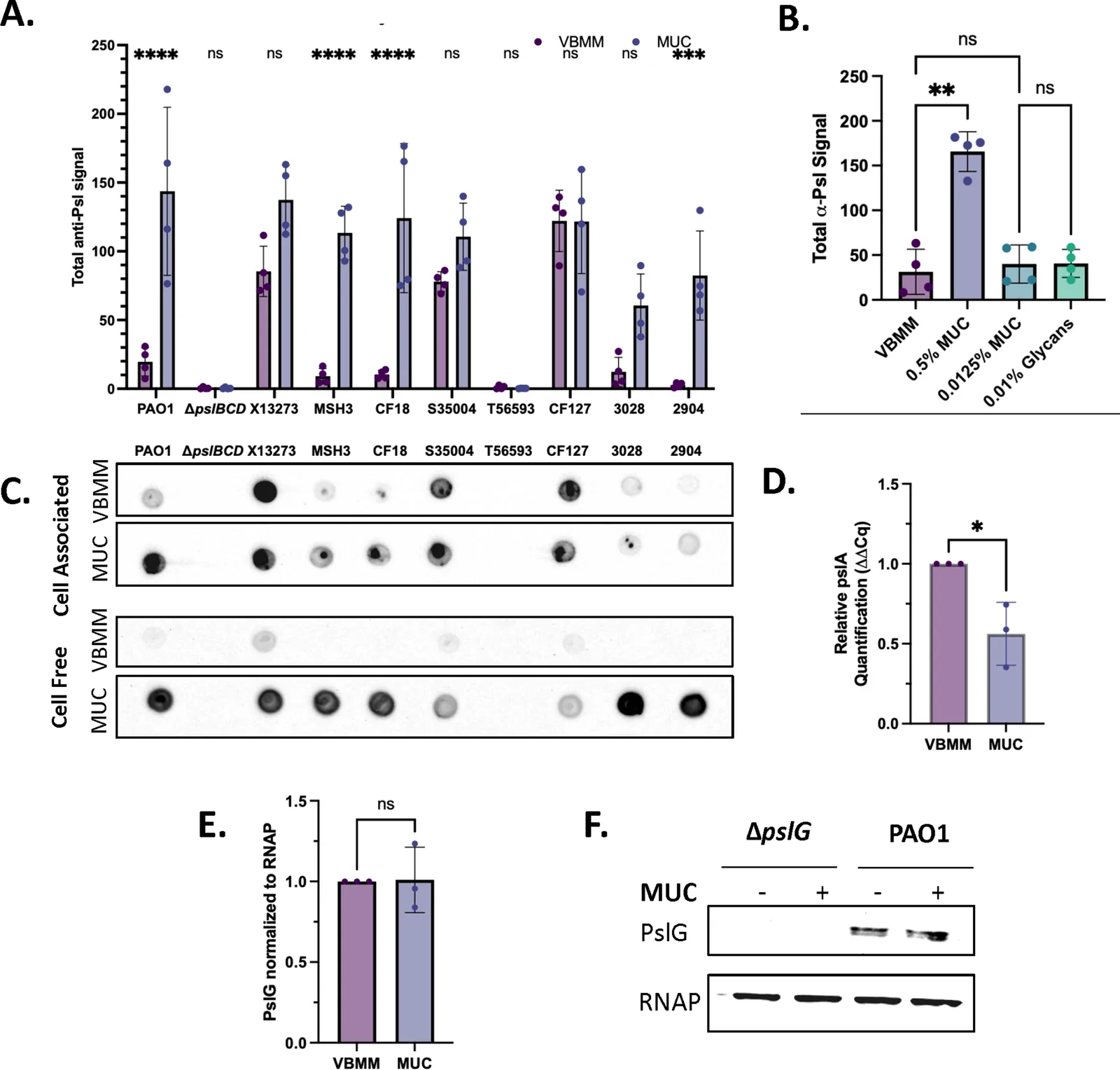
Mucin stimulates Psl production. (**A**) Total Psl signal from a cohort of environmental and clinical isolates after exposure to Volger-B minimal media (VBMM) ± 0.5% (wt/vol) MUC. Stationary phase cells were normalized to in VBMM ± 0.5% (wt/vol) MUC for 1 h shaking at 37°C before plating for CFU. Cells were then pelleted to obtain cell-free and cell-associated fractions of Psl before dot blotting. Samples were spotted in triplicate, and experiments were repeated three times. Representative dot blot of mucin-treated cells showing cell-free and cell-associated fractions (below). *N* = 3 and *n* = 3. Significance was determined via RM two-way analysis of variance (ANOVA) with Šidák’s multiple comparison test, *, *P* < 0.05; ****, *P* < 0.0001; and ns, not significant. (**B**) Total Psl signal of PAO1 exposed to 0.5% (wt/vol) MUC, 0.0125% (wt/vol) MUC, or 0.01% (wt/vol) glycans. *N* = 3 and *n* = 3. Significance was determined via RM two-way ANOVA with Geisser-Greenhouse corrections and Tukey’s multiple comparisons test. (**C**) Representative cell-free and cell-associated Psl dot blots of isolate strains. (**D**) RNA of MUC-treated cells for 1 h was isolated, DNase treated, and reverse transcribed into cDNA before running qPCR for *pslA* transcripts. ∆∆Cq values were calculated against the housekeeping gene, *recA. n* = 3. Significance was determined via an unpaired *t*-test, *, *P* < 0.05; and ns, not significant. (**E**) Lysates of MUC-treated cells (1 h) were probed with ɑ-PslG and ɑ-βRNAP antibodies. The amount of PslG relative to βRNAP was determined using FIJI. *n* = 3. Significance was determined via an unpaired *t*-test. (**F**) Representative western blot for PslG against the loading control.

While pili-deficient cells do not produce Psl trails in mucin, an increase in Psl signal around Δ*pilA* cells was observed upon mucin addition (Fig. 4A). To determine whether mucin elevates Psl production, planktonic cultures of PAO1 or Δ*pslBCD* were exposed to mucin for 1 h and evaluated for Psl levels using a monoclonal Psl antibody cocktail (32). Compared to media alone, mucin increased both cell-free and cell-associated Psl, independent of growth and in a concentration-dependent manner, resulting in an increase in total Psl produced in PAO1 but not Δ*pslBCD* (Fig. 5A and C; Fig. S6 and S8). We examined how universal this phenotype is amongst a cohort of *P. aeruginosa* isolates of various origins (Table 1) and found that mucin increased Psl production in most Psl-producing isolates but to varying degrees, including isolates derived from non-mucosal infection sites (MSH3, X13273, and S35004; Fig. 5A and C). One isolate, T56593, from an ear infection, had no increase in Psl after mucin exposure, but this isolate also did not produce Psl under control conditions. Furthermore, no significant increase was observed when a lower concentration of glycans was tested for inducing Psl production (Fig. 5B). A comparable level of mucin (0.0125% [wt/vol]) to the glycan concentration used (0.01% [wt/vol]) was also unable to significantly impact Psl levels as detectable via immunoblotting (Fig. 5B).

**TABLE 1:** Strains and environmental and clinical isolate sources

Strain	Source/description	Reference(s)
X13273	Blood isolate; Seattle, WA	(36, 37)
MSH3	Environmental (water); Spirit Lake, WA	(36)
CF18	Cystic fibrosis; Jackson, MS	(36)
S35004	Blood isolate; Seattle, WA	(36)
T56593	Ear infection; Wake Forest University, NC	(38)
CF127	Cystic fibrosis; Denver, CO	(36)
3028	Cystic fibrosis (mucoid); Columbus, OH	(39)
2904	Cystic fibrosis (mucoid); Columbus, OH	(39, 40)
PAO1 (mPAO1)	WT *P. aeruginosa*	(41)
PAO1Δ*pslBCD*	Psl-deficient mutant	(42)
PAO1Δ*pilA*	Pilin-deficient mutant	(43)
PAO1Δ*pslG*	Psl hydrolase deficient mutant	(44)
PAO1pBAD-Ppsl-Δ*pel*	Psl promoter replaced with arabinose-inducible promoter (pBAD). Also Pel deficient.	This study
PAO1pBAD-Ppsl*-*Δ*pel* Δ*wspF*	WspF deletion mutant. High c-di-GMP background with Psl promoter replaced with pBAD. Also Pel deficient.	This study
PAO1pBAD-Ppsl*-*Δ*pel* PDE2133	PDE2133 expressing strain on a plasmid (pUCP18). Low c-di-GMP background with Psl promoter replaced with pBAD. Also Pel deficient.	This study

To determine whether the increase in Psl was through a mucin-dependent transcriptional response targeting expression of the *psl* operon, qPCR was performed on planktonic cells exposed to mucin for 1 h (Table 2). Expression of *pslA* did not increase in mucin and instead trended downward (Fig. 5D). A similar decrease in *psl* operon transcripts was observed in an RNA-seq study after exposure to purified MUC5AC mucin (5). There is one known post-transcriptional regulation mechanism of the *psl* operon, RsmA, which binds to the 5′ untranslated region of *psl* mRNA to block ribosome binding and subsequent translation. Therefore, we next determined levels of a Psl protein, PslG, via western blot after the same mucin exposure period to determine if mucin is impacting post-transcriptional regulation mechanisms, but PslG levels remain unaltered in mucin compared to the medium-alone control (Fig. 5E and F) (45). Taken together, these data suggest that mucin is impacting Psl levels independent of transcriptional or translational regulation mechanisms. Given the extensive transcriptional and post-transcriptional regulation of the *psl* operon, the rapid increase in Psl levels without corresponding increases in *pslA* transcripts or PslG protein suggests engagement of an unrecognized, non-genetic regulatory layer that is responsive to environmental context.

**TABLE 2:** qPCR primer list

Primer	Sequence
pilA5	ACGGAACAGGATGTAGAGGTCGAA
pilA4	TGCACAAGATCAAGAAACGCGTGG

### c-di-GMP post-translationally regulates Psl production, but mucin does not alter c-di-GMP levels

**Fig 6:**
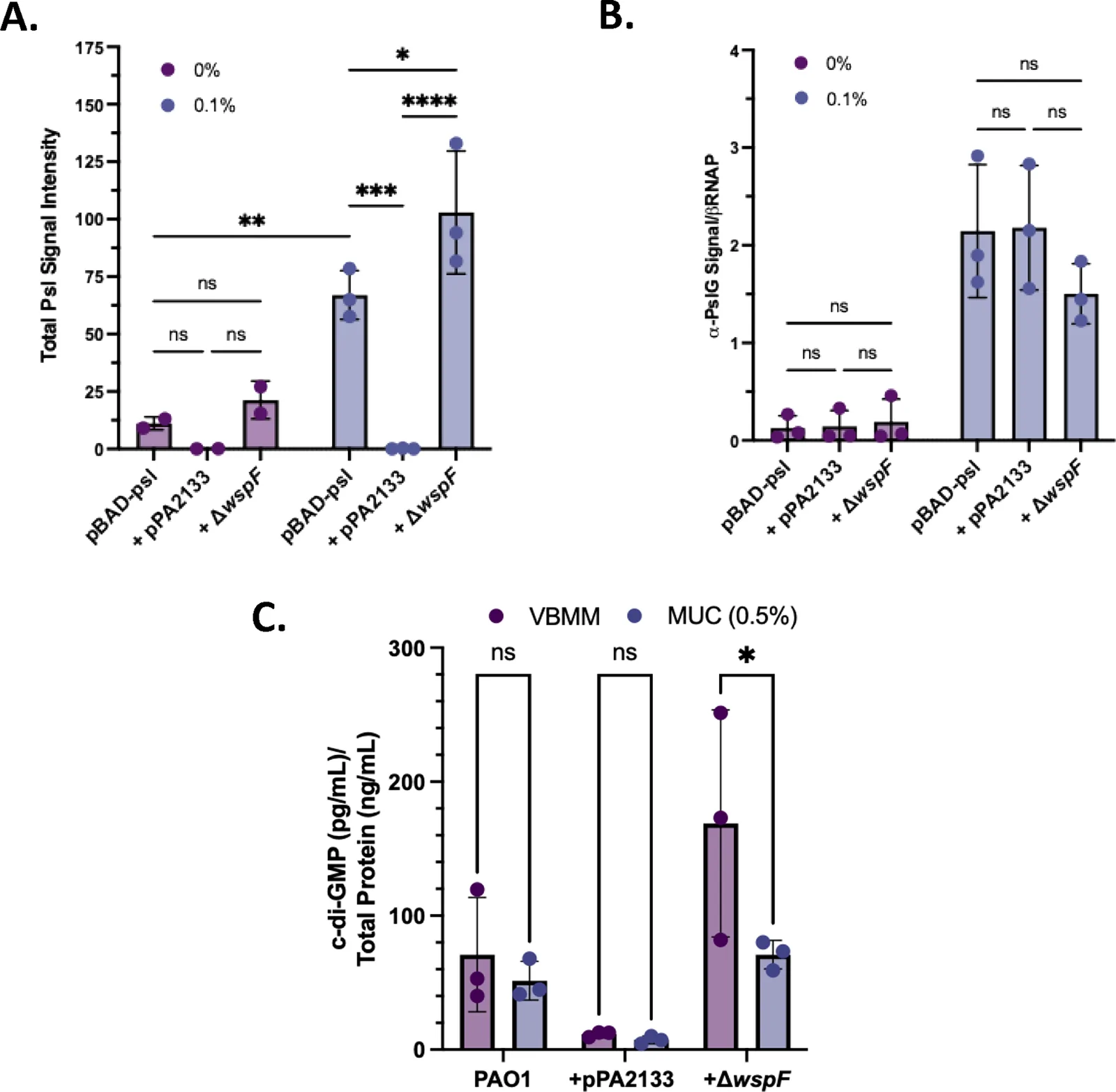
Psl production is post-translationally regulated by c-di-GMP. (**A**) Psl dot blots of *psl* promoter replaced with p*BAD-psl* strains with low (PA2133), unchanged (p*BAD-psl*), and high (Δ*wspF*) c-di-GMP backgrounds under no induction (blue) and induction (0.1%) conditions (purple). *N* = 3, *n* = 2 for 0%, and *n* = 3 for 0.1%. Significance determined via mixed-effects model with Tukey’s multiple comparisons test, *, *P* < 0.05; ****, *P* < 0.001; and ns, not significant. (**B**) Western blots of PslG relative to β-RNAP in these strain backgrounds under the same conditions. *n* = 3. Significance was determined via ordinary two-way analysis of variance (ANOVA) with Šidák’s multiple comparison test. (**C**) Cells exposed to Volger-B minimal media (VBMM) ± 0.5% (wt/vol) MUC for 1 h were harvested, lysed, and used to perform a c-di-GMP enzyme-linked immunosorbent assay (ELISA). c-di-GMP levels are plotted relative to total protein as determined via Bradford assay. *n* = 3. Significance determined via ordinary two-way ANOVA with Šidák’s multiple comparison test, *, *P* < 0.05; and ns, not significant.

As mucin induction of Psl did not appear to be due to transcriptional or translational regulation, we sought to explore post-translation regulation mechanisms. At this time, no known post-translational mechanisms have been identified for Psl production, but previous work has identified the secondary messenger, c-di-GMP, as a post-translational regulator of alginate and Pel synthesis (38, 46). c-di-GMP is also a transcriptional regulator of the *psl* operon via FleQ; therefore, to examine c-di-GMP effects on Psl production independent of any transcriptional regulation, we developed a strain where the *psl* promoter was replaced with an arabinose-inducible p*BAD* promoter, thus uncoupling *psl* transcription from FleQ or c-di-GMP (32, 47). For low c-di-GMP conditions, we overexpressed the phosphodiesterase (PDE) PA2133, which degrades c-di-GMP. For high c-di-GMP conditions, we used a Δ*wspF* mutant, which results in constitutive c-di-GMP synthesis (48). Under these conditions, we observed a clear increase in Psl production under high c-di-GMP conditions despite uncoupling c-di-GMP from *psl* transcription. Additionally, c-di-GMP appeared to be required for Psl synthesis, as the PDE-expressing strain produced no detectable levels of Psl (Fig. 6A). To confirm that there were no unknown post-transcriptional effects of c-di-GMP, we also determined no changes in PslG protein levels under low or high c-di-GMP conditions (Fig. 6B). Together, these data suggest that c-di-GMP can additionally regulate Psl synthesis post-translationally, a finding not previously described.

We therefore hypothesized that mucin may be increasing Psl production post-translationally through rapid modulation of c-di-GMP levels. To test this, cells were exposed to mucin for 1 h before lysing, and c-di-GMP levels were quantified via enzyme-linked immunosorbent assay (ELISA). A Δ*wspF* mutant and the PA2133 PDE-expressing strain served as controls. Surprisingly, c-di-GMP levels of mucin-treated WT cells were comparable to those of cells in media alone (Fig. 6C). This suggests that mucin is increasing Psl production through an unknown mechanism that is independent of any changes in transcription, translation, and global levels of c-di-GMP.

### Excess Psl in mucin protects against antimicrobials

**Fig 7:**
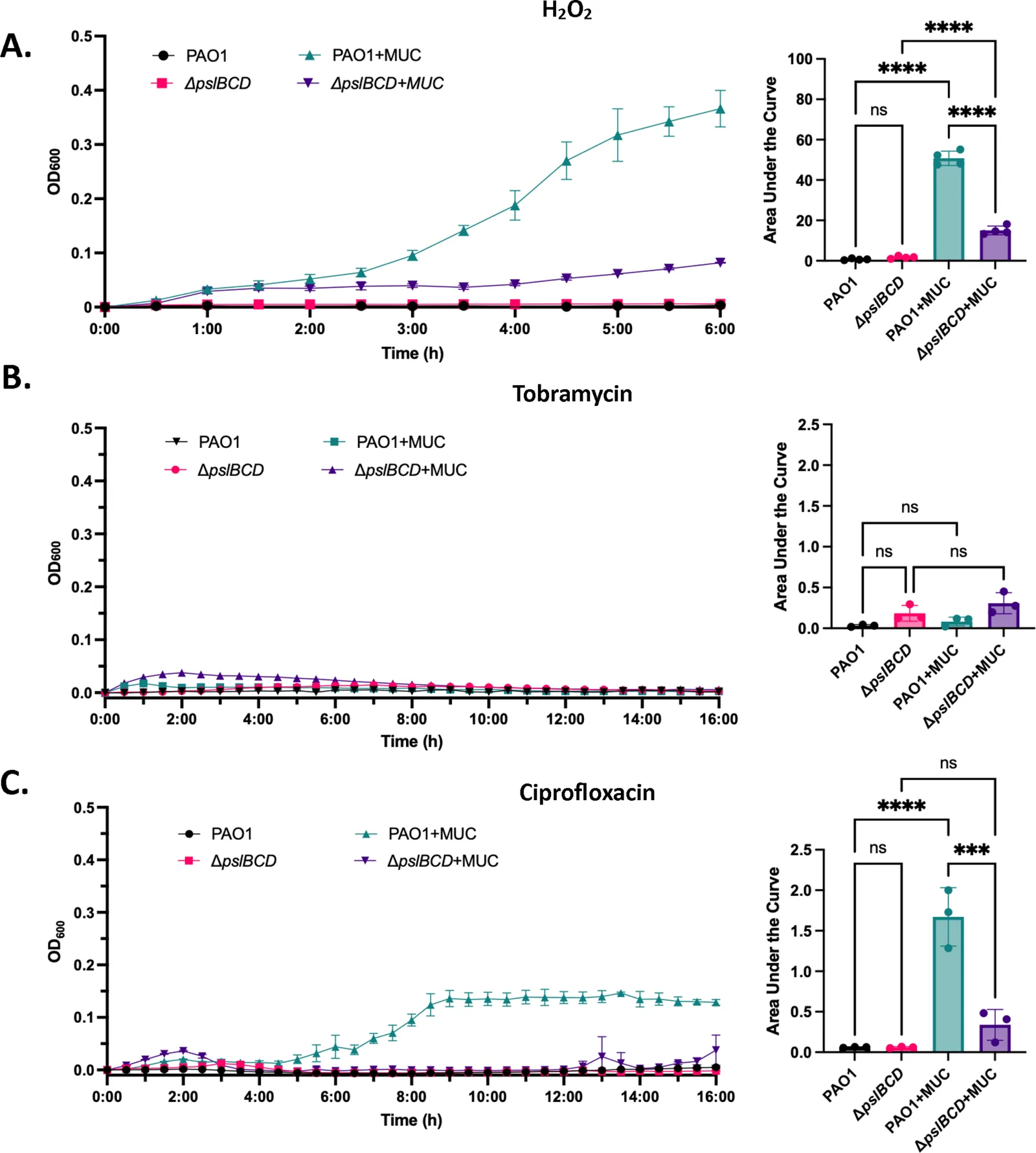
Mucin-induced Psl provides protection against select antimicrobials. Left: PAO1 or Δ*pslBCD* cells were pre-exposed to 0.5% MUC for 1 h before adding MIC H_2_O_2_ (2 mM) (**A**), tobramycin (4 μg/mL) (**B**), or ciprofloxacin (1 μg/mL) (**C**). OD_600_ was measured for 6 or 16 h. A representative growth curve is displayed. Right: changes in OD were quantified as area under the curve. *N* = 2 and *n* = 3. Significance determined via ordinary one-way analysis of variance with Tukey’s multiple comparisons test, ***, *P* < 0.001; ****, *P* < 0.0001; and ns, not significant.

Depending on strain background and growth conditions, Psl provides protection against several antimicrobials such as tobramycin, colistin, polymyxin B, ciprofloxacin, and H_2_O_2_, depending on whether *P. aeruginosa* is in the biofilm or planktonic state (21, 23, 24). We hypothesized that the mucin-induced increase in Psl would afford some degree of protection against these antimicrobials. To address this, PAO1 or Δ*pslBCD* cells were either untreated or exposed to mucin for 1 h. Following this, select antimicrobials at 1× MIC were added, and microbial growth was measured for 6–16 h. We found that mucin-exposed PAO1 was able to grow in the presence of H_2_O_2_ and ciprofloxacin while untreated cells failed to grow (Fig. 7A and C; Fig. S10). However, exposure to mucin did not allow PAO1 to grow in the presence of tobramycin (Fig. 7B; Fig. S10). This suggests that mucin-stimulated Psl production provides *P. aeruginosa* with a fitness advantage in the presence of certain antimicrobials. Additionally, we observed that mucin provides modest growth against H_2_O_2_ independent of Psl production, as a Δ*pslBCD* mutant can grow slightly in mucin compared to media alone, but viable cell plating did not reveal any significant differences in CFU, unlike for PAO1 in H_2_O_2_ (Fig. 7A; Fig. S10A).

## DISCUSSION

In this study, we show how exposure to a specific component of mucus, the glycoprotein mucin, promotes surface exploration and Psl production in *P. aeruginosa*. This work is consistent with the study by Nolan et al., where they demonstrated that commercial mucin (0.05% [wt/vol]) stimulated twitching motility in the non-Psl-producing *P. aeruginosa* lab strains PAK and PA14 (49). Nolan et al. attributed this effect to protease activity releasing oligopeptides, as the addition of a protease inhibitor reduced mucin-stimulated twitching motility. However, we observed that the glycan structures of mucin alone are also able to stimulate twitching motility, which then contributes to the formation of Psl trails (Fig. 3B and 4C). Consistent with this interpretation, the ability of both partially degraded industrial mucin and its released O-glycan pool to promote Psl trail formation suggests that *P. aeruginosa* responds to glycan-associated features that are enriched or more accessible when mucins are structurally compromised, rather than to intact mucin polymers or individual monosaccharides. Thus, our work suggests an oligopeptide-independent signal for *P. aeruginosa* that promotes twitching motility as well as induces formation of Psl trails. An unanswered paradox that remains is how mucin promotes Psl trails while simultaneously reducing adhered cell numbers and hindering subsequent biofilm formation. Recent work from Schmidt et al. (50) has perhaps provided mechanistic insight into how cells interact with Psl trails via the cell-associated adhesin, CdrA, to promote biofilm formation. One possibility is that mucin is interfering with CdrA-Psl interactions, which would then reduce the subsequent mechano-sensing signals that are transmitted via pili to induce the biofilm state (50). While CdrA-mucin binding has yet to be demonstrated, mucin contains similar sugar residues like GalNAc, fucose, and mannose that CdrA has been suggested to interact with reference 11.

Additionally, we show mucin exposure stimulates Psl production, even in the absence of a surface (Fig. 5A). Our data further indicate that altered mucin environments bias twitching motility and Psl accumulation independent of one another. Twitching motility is enhanced even in Psl-deficient strains, as had been demonstrated by Nolan et al. with reference strain PA14, while increased Psl levels are observed in non-twitching mutants (Δ*pilA*), demonstrating that these responses are separable adaptive behaviors rather than a single linear pathway (Fig. 4A; Fig. S6) (49). This decoupling underscores the role of mucus context in shaping bacterial behavioral states rather than activating a single dedicated signaling cascade. In support of this, mucin stimulates twitching motility in the naturally Psl-deficient strain, PA14, as well as *psl* mutant strains of PAO1 (Fig. S4 and S9) (49). Furthermore, glycans were unable to increase total Psl levels but were seen to stimulate Psl trails (Fig. 3B and 5B). This implies that an increase in Psl production is not needed for Psl trail formation, and native levels of Psl are sufficient for trail formation if twitching motility is induced. A comparative level of mucin was also unable to stimulate significant Psl production, with additional work showing Psl stimulation is not achieved until higher concentrations, suggesting that increased glycan levels might be necessary to induce Psl production (Fig. 4B; Fig. S5).

Psl is transcriptionally regulated through LasR, RpoS, AmrZ, and FleQ and post-transcriptionally through RsmA (45, 47, 51). However, mucin increased Psl levels, both cell associated and cell free, independent of any of these known mechanisms, with no increase in *pslA* transcripts or PslG protein observed (Fig. 5E and F). Pel and alginate synthesis are regulated post-translationally by c-di-GMP levels via c-di-GMP binding pockets on PelD and Alg44, respectively (38, 46). No c-di-GMP binding pocket has been identified for any of the Psl synthesis proteins, but we have now shown that under elevated c-di-GMP conditions (Δ*wspF*), Psl levels are increased, and this effect is reversed with lowered c-di-GMP conditions (Fig. 6A). While this does not prove c-di-GMP is directly binding with components of the Psl machinery, it does indicate that c-di-GMP influences Psl production independent of transcriptional or translational regulation mechanisms (Fig. 6A and B). While no measurable changes in global c-di-GMP levels were observed in mucin, it could be possible that mucin is modulating localized pools of c-di-GMP, as observed for flagella switching, which would be otherwise overlooked in total cell lysate measurements (52, 53). An alternative explanation for increased Psl levels in mucin could be through posttranscriptional regulation by substrate competition. Previous work from our lab and others has shown that the sugar precursors necessary for Psl synthesis are shared by additional pathways like alginate, rhamnolipid, and lipopolysaccharide (LPS) synthesis (44, 54). Furthermore, additional studies have revealed that manipulating levels of these end products alters Psl levels, so it is possible that mucin is increasing Psl production through modulating sugar precursor distribution (55, 56).

Previous work has established that Psl provides protection from various antimicrobials, regardless of whether the cell is encapsulated in a biofilm matrix or in a planktonic state (21, 57). Additional studies have also demonstrated that mucin reduces the efficacy of certain antimicrobials, mostly through mucin-antibiotic binding interactions (58, 59). Here, we provide evidence that in addition to passive physicochemical effects on antibiotic efficacy, altered mucin environments appear to bias *P. aeruginosa* toward self-protective behaviors through increased Psl accumulation. This effect is particularly evident for ciprofloxacin and hydrogen peroxide, where mucin-exposed wild-type cells, but not Δ*pslBCD* mutants, exhibit antimicrobial tolerance (Fig. 7A and C). Previously, mucin had been shown to protect PAO1 from ciprofloxacin killing independent of mucin-antibiotic binding, and our current work suggests that this may be due to Psl (59). A Psl-producing strain also displays significantly increased growth in the presence of H_2_O_2_ compared to a Δ*pslBCD* mutant, with mucin alone appearing to provide some level of protection against H_2_O_2_ as Δ*pslBCD* grows to a modest degree in mucin compared to no growth in media alone (Fig. 7A). These findings suggest that antibiotic exposure within degraded mucus environments may inadvertently select for matrix-reinforced persistence, providing a mechanistic explanation for treatment failure despite susceptibility in standard *in vitro* assays.

Overall, our study supports a growing body of work demonstrating that mucin alters *P. aeruginosa* virulence behaviors. We now extend these findings by showing that mucin-mediated behavioral changes occur post-translationally at a rapid timescale (<1 h). Furthermore, we demonstrate, for the first time, that Psl is subject to post-translational regulation by c-di-GMP and that mucin stimulates Psl production independent of previously established transcriptional and post-transcriptional pathways. These findings help reconcile observations that Psl transcripts decrease upon mucin exposure in transcriptomic studies, yet Psl is consistently detected in infection samples (8, 26). Given that Psl provides critical protection against antimicrobials, understanding how a key component of the mucosal environment, mucin, increases Psl levels can provide further insight into *P. aeruginosa* survival strategies during host infection. More broadly, these results highlight the importance of modeling disease-relevant mucus states when evaluating bacterial virulence and therapeutic efficacy and suggest that preserving mucus integrity may be an underappreciated strategy for limiting chronic infection persistence.

## MATERIALS AND METHODS

### Growth and strain conditions

Strains were cultured with shaking overnight in lysogeny broth (LB) for approximately 16 h with appropriate antibiotic concentrations when needed for reporter constructs. Where appropriate, plasmid constructs were also incubated with 300 μg/mL carbenicillin, and induction of P_BAD_-psl cultures was with 1% arabinose. Cultures were spun, washed, and concentrated to 2 OD before diluting to 0.05 OD in Volger-B minimal media (VBMM) ± 0.5% (wt/vol) porcine gastric mucin (Sigma), MUC5AC purified from porcine gastric, carboxymethylcellulose (Thermofischer), or 0.01% (wt/vol) porcine gastric mucin glycans, core 1 glycan (CAS 20972-29-6)*,* or a pool of monosaccharides containing 0.01% total (wt/vol) of equal amounts galactose, N-acetylglucosamine, N-acetylgalactosamine, fucose, and sialic acid (Sigma). Commercial preparations of mucin were resuspended overnight, rocking at 4°C, before centrifuging to remove any insoluble components. The concentration of mucin-glycans was chosen based on dose-response results for Psl trail presentation as well as previously published results indicating 0.01% (wt/vol) mucin was sufficient for measurable effects (8). Due to the technical limitations in preparing large-scale samples of mucin-glycans, the lowest concentration of glycans that generated measurable effects was used. Samples were added to uncoated Ibidi μ-Slide VI 0.1 channel slides (Item No. 80661-90) and observed under 60× wide-field microscopy with a Ti2 Nikon for 1 h every 2.5 or 5 min. Cell tracking was performed via FIJI extension TrackMate with between 150 and 400 cells analyzed per replicate (60). Image sequences were drift adjusted via the 3D-Drift Correction package before analysis (61). Post-incubation period, loosely adhered cells were removed via 3× PBS washes. The remaining adhered cells were fixed with 4% PFA.

### Psl staining

Fixed cells were blocked with 3% bovine serum albumin before adding monoclonal cocktail (AstraZeneca, 1:3,000) of α-Psl, followed by secondary α-human IgG conjugated to Alexafluor 647 (1:1,000). Samples were imaged via wide-field microscopy for Psl trails.

### Psl quantification (dot blots)

Overnight cultures grown in LB were normalized to 2 OD before resuspending in an equal volume of 0.5% (wt/vol) of MUC or VBMM. Samples were incubated for 1 h, shaking at 37°C. Post-incubation, samples were plated for CFU before dot blotting. Briefly, cell-associated and cell-free fractions of Psl were separated through pelleting. The supernatant was filter sterilized through a 0.22 μm filter to give a cell-free fraction of Psl. The pellet was then resuspended in 0.5 M EDTA and boiled at 100°C for 10 min before pelleting to give a cell-associated fraction of Psl. Two microliters of each fraction was pipetted onto a nitrocellulose (0.2 μm) membrane and air-dried for 10 min, before being blocked with 5% skim milk in TBST (0.1% Tween 20) rocking for 1 h. A primary antibody of 1:3,000 α-Psl was added for 1 h before washing 3× and adding α-human IgG-HRP at 1:5,000 for another hour before washing again 3×. Blots were imaged via BioRad Chemidoc Imaging System. Images were analyzed via FIJI.

### pslA qPCR

RNA isolation was performed with a modified procedure on overnight cultures of PAO1 after 1 h incubation with VBMM or 0.5% MUC with Qiagen RNeasy kit. DNAse treatments were performed with RNase-free DNase I (Thermo Scientific) before cDNA synthesis with iScript Reverse Transcription Supermix for RT-qPCR (BioRad). Samples were stored at −20°C until qPCR was performed. Briefly, qPCRs were set up with SYBR Green with either rpoD or recA as housekeeping gene control and run on BioRad CFX Connect Real-Time System. Results were analyzed to give a ΔΔCt value.

### PslG western blot

Cells were exposed to VBMM or 0.5% (wt/vol) MUC for 1 h before washing and lysing via sonication. Protein quantification was performed via Bradford assay. Approximately 15 μg samples were then run on a 12% SDS-PAGE gel for 45 min with stacking at 70 V, 1.5 h resolving at 120 V before wet transfer at 0.15 Amp for 1 h on nitrocellulose membrane (0.2 μm). Membranes were blocked for 1 h with 5% skim milk in TBST (0.1% Tween 20) before overnight primary incubation with PslG (1:5,000) or β-RNAP (1:5,000). Membranes were washed 3× with TBST (0.1% Tween 20) before secondary incubation with anti-rabbit IgG-HRP (1:5,000) for 1 h. Membranes were washed 3× with TBST (0.1% Tween) before imaging with ECL Western Blotting Detection Reagents (Cytiva) developer on BioRad Chemidoc Imaging System. Images were analyzed with FIJI, and anti-PslG signal was normalized to β-RNAP.

### c-di-GMP ELISA

Cultures grown overnight in LB were washed and resuspended in VBMM ± 0.5% MUC (wt/vol) before incubating for 1 h at 37°C shaking. Post-incubation, cells were spun down and resuspended in 100 μL of BPER before bead-beating with 0.2 μm beads for 2 cycles at 6,600 rpm in a Bertin Precellys24 Touch to lyse cells. The supernatant was then taken for protein quantification via Bradford assay or ELISA via Cayman Chemical Company cyclic-di-GMP ELISA kit (Item No. 501780).

### Antimicrobial testing

Cultures were grown overnight in LB before diluting back and allowing regrowth to the stationary phase (OD ~1). Cultures were then centrifuged and resuspended in VBMM ± 0.5% (wt/vol) MUC and incubated shaking at 37°C for 1 h. Cultures were then subdivided and appropriate antibiotics at MIC concentrations added before transferring to a 96-well plate and recording OD_600_ for 6–16 h shaking at 37°C. MICs were determined as the concentration of antibiotic that resulted in no visible growth.

## SUPPLEMENTAL MATERIAL

Supplemental figures mbio.01445-26-s0001.docx Figures S1 to S10.

Video S1 mbio.01445-26-s0002.mp4 Twitching videos of PAO1 in medium alone (VBMM) or in 0.5% (wt/vol) MUC.

ASM does not own the copyrights to Supplemental Material that may be linked to, or accessed through, an article. The authors have granted ASM a non-exclusive, world-wide license to publish the Supplemental Material files. Please contact the corresponding author directly for reuse.

## References

[1] Rossi E, La Rosa R, Bartell JA, Marvig RL, Haagensen JAJ, Sommer LM, Molin S, Johansen HK. 2021. *Pseudomonas aeruginosa* adaptation and evolution in patients with cystic fibrosis. Nat Rev Microbiol 19:331–342. doi:10.1038/s41579-020-00477-5 33214718

[2] Newman JN, Floyd RV, Fothergill JL. 2022. Invasion and diversity in *Pseudomonas aeruginosa* urinary tract infections. J Med Microbiol 71:001458. doi:10.1099/jmm.0.001458 35275806 PMC9176269

[3] Badger-Emeka L, Emeka P, Thirugnanasambantham K, Alatawi AS. 2024. The role of *Pseudomonas aeruginosa* in the pathogenesis of corneal ulcer, its associated virulence factors, and suggested novel treatment approaches. Pharmaceutics 16:1074. doi:10.3390/pharmaceutics16081074 39204419 PMC11360345

[4] Matheus GG, Chamoun MN, Khosrotehrani K, Sivakumaran Y, Wells TJ. 2025. Understanding the pathophysiology of *Pseudomonas aeruginosa* colonization as a guide for future treatment for chronic leg ulcers. Burns Trauma 13:tkae083. doi:10.1093/burnst/tkae083 39830194 PMC11741523

[5] Fahy JV, Dickey BF. 2010. Airway mucus function and dysfunction. N Engl J Med 363:2233–2247. doi:10.1056/NEJMra0910061 21121836 PMC4048736

[6] Takagi J, Aoki K, Turner BS, Lamont S, Lehoux S, Kavanaugh N, Gulati M, Valle Arevalo A, Lawrence TJ, Kim CY, et al.. 2022. Mucin O-glycans are natural inhibitors of *Candida albicans* pathogenicity. Nat Chem Biol 18:762–773. doi:10.1038/s41589-022-01035-1 35668191 PMC7613833

[7] Alemka A, Whelan S, Gough R, Clyne M, Gallagher ME, Carrington SD, Bourke B. 2010. Purified chicken intestinal mucin attenuates *Campylobacter jejuni* pathogenicity *in vitro*. J Med Microbiol 59:898–903. doi:10.1099/jmm.0.019315-0 20466838

[8] Wheeler KM, Cárcamo-Oyarce G, Turner BS, Dellos-Nolan S, Co JY, Lehoux S, Cummings RD, Wozniak DJ, Ribbeck K. 2019. Mucin glycans attenuate the virulence of *Pseudomonas aeruginosa* in infection. Nat Microbiol 4:2146–2154. doi:10.1038/s41564-019-0581-8 31611643 PMC7157942

[9] Co JY, Cárcamo-Oyarce G, Billings N, Wheeler KM, Grindy SC, Holten-Andersen N, Ribbeck K. 2018. Mucins trigger dispersal of *Pseudomonas aeruginosa* biofilms. NPJ Biofilms Microbiomes 4:23. doi:10.1038/s41522-018-0067-0 30323945 PMC6180003

[10] Ricciuto J, Heimer SR, Gilmore MS, Argüeso P. 2008. Cell surface O-glycans limit *Staphylococcus aureus* adherence to corneal epithelial cells. Infect Immun 76:5215–5220. doi:10.1128/IAI.00708-08 18794288 PMC2573382

[11] Kavanaugh NL, Zhang AQ, Nobile CJ, Johnson AD, Ribbeck K. 2014. Mucins suppress virulence traits of *Candida albicans*. mBio 5:e01911-14. doi:10.1128/mBio.01911-14 25389175 PMC4235211

[12] Xia B, Royall JA, Damera G, Sachdev GP, Cummings RD. 2005. Altered O-glycosylation and sulfation of airway mucins associated with cystic fibrosis. Glycobiology 15:747–775. doi:10.1093/glycob/cwi061 15994837

[13] Wagner C.E., Wheeler KM, Ribbeck K. 2018. Mucins and their role in shaping the functions of mucus barriers. Annu Rev Cell Dev Biol 34:189–215. doi:10.1146/annurev-cellbio-100617-062818 30296390 PMC11906035

[14] Wagner Caroline E., Krupkin M, Smith-Dupont KB, Wu CM, Bustos NA, Witten J, Ribbeck K. 2023. Comparison of physicochemical properties of native mucus and reconstituted mucin gels. Biomacromolecules 24:628–639. doi:10.1021/acs.biomac.2c01016 36727870

[15] Henderson AG, Ehre C, Button B, Abdullah LH, Cai L-H, Leigh MW, DeMaria GC, Matsui H, Donaldson SH, Davis CW, et al.. 2014. Cystic fibrosis airway secretions exhibit mucin hyperconcentration and increased osmotic pressure. J Clin Invest 124:3047–3060. doi:10.1172/JCI73469 24892808 PMC4072023

[16] Batson BD, Zorn BT, Radicioni G, Livengood SS, Kumagai T, Dang H, Ceppe A, Clapp PW, Tunney M, Elborn JS, et al.. 2022. Cystic fibrosis airway mucus hyperconcentration produces a vicious cycle of mucin, pathogen, and inflammatory interactions that promotes disease persistence. Am J Respir Cell Mol Biol 67:253–265. doi:10.1165/rcmb.2021-0359OC 35486871 PMC9348562

[17] Ramsey KA, Chen ACH, Radicioni G, Lourie R, Martin M, Broomfield A, Sheng YH, Hasnain SZ, Radford-Smith G, Simms LA, et al.. 2020. Airway mucus hyperconcentration in non-cystic fibrosis bronchiectasis. Am J Respir Crit Care Med 201:661–670. doi:10.1164/rccm.201906-1219OC 31765597 PMC7068838

[18] Borlee BR, Goldman AD, Murakami K, Samudrala R, Wozniak DJ, Parsek MR. 2010. *Pseudomonas aeruginosa* uses a cyclic-di-GMP-regulated adhesin to reinforce the biofilm extracellular matrix. Mol Microbiol 75:827–842. doi:10.1111/j.1365-2958.2009.06991.x 20088866 PMC2847200

[19] Jones CJ, Wozniak DJ. 2017. Psl produced by mucoid *Pseudomonas aeruginosa* contributes to the establishment of biofilms and immune evasion. mBio 8:e00864-17. doi:10.1128/mBio.00864-17 28634241 PMC5478896

[20] Ma L, Jackson KD, Landry RM, Parsek MR, Wozniak DJ. 2006. Analysis of *Pseudomonas aeruginosa* conditional psl variants reveals roles for the psl polysaccharide in adhesion and maintaining biofilm structure postattachment. J Bacteriol 188:8213–8221. doi:10.1128/JB.01202-06 16980452 PMC1698210

[21] Billings N, Millan M, Caldara M, Rusconi R, Tarasova Y, Stocker R, Ribbeck K. 2013. The extracellular matrix component Psl provides fast-acting antibiotic defense in *Pseudomonas aeruginosa* biofilms. PLoS Pathog 9:e1003526. doi:10.1371/journal.ppat.1003526 23950711 PMC3738486

[22] Byrd MS, Pang B, Mishra M, Swords WE, Wozniak DJ. 2010. The *Pseudomonas aeruginosa* exopolysaccharide Psl facilitates surface adherence and NF-kappaB activation in A549 cells. mBio 1:e00140-10. doi:10.1128/mBio.00140-10 20802825 PMC2925078

[23] da Cruz Nizer WS, Allison KN, Adams ME, Vargas MA, Ahmed D, Beaulieu C, Raju D, Cassol E, Howell PL, Overhage J. 2024. The role of exopolysaccharides Psl and Pel in resistance of *Pseudomonas aeruginosa* to the oxidative stressors sodium hypochlorite and hydrogen peroxide. Microbiol Spectr 12:e00922-24. doi:10.1128/spectrum.00922-24 39194290 PMC11448232

[24] Grossich R, Lemos Vilches M, Costa CS, Pezzoni M. 2023. Role of Pel and Psl polysaccharides in the response of *Pseudomonas aeruginosa* to environmental challenges: oxidative stress agents (UVA, H2O2, sodium hypochlorite) and its competitor *Staphylococcus aureus*. Microbiology 169:001301. doi:10.1099/mic.0.001301 36757866 PMC10197878

[25] Mishra M, Byrd MS, Sergeant S, Azad AK, Parsek MR, McPhail L, Schlesinger LS, Wozniak DJ. 2012. *Pseudomonas aeruginosa* Psl polysaccharide reduces neutrophil phagocytosis and the oxidative response by limiting complement-mediated opsonization. Cell Microbiol 14:95–106. doi:10.1111/j.1462-5822.2011.01704.x 21951860 PMC4466118

[26] Jennings LK, Dreifus JE, Reichhardt C, Storek KM, Secor PR, Wozniak DJ, Hisert KB, Parsek MR. 2021. *Pseudomonas aeruginosa* aggregates in cystic fibrosis sputum produce exopolysaccharides that likely impede current therapies. Cell Rep 34:108782. doi:10.1016/j.celrep.2021.108782 33626358 PMC7958924

[27] Zegans ME, DiGiandomenico A, Ray K, Naimie A, Keller AE, Stover CK, Lalitha P, Srinivasan M, Acharya NR, Lietman TM. 2016. Association of biofilm formation, Psl exopolysaccharide expression, and clinical outcomes in *Pseudomonas aeruginosa* keratitis: analysis of isolates in the steroids for corneal ulcers trial. JAMA Ophthalmol 134:383–389. doi:10.1001/jamaophthalmol.2015.5956 26846404

[28] Morris AJ, Yau YCW, Park S, Eisha S, McDonald N, Parsek MR, Howell PL, Hoffman LR, Nguyen D, DiGiandomenico A, et al.. 2022. *Pseudomonas aeruginosa* aggregation and Psl expression in sputum is associated with antibiotic eradication failure in children with cystic fibrosis. Sci Rep 12:21444. doi:10.1038/s41598-022-25889-6 36509824 PMC9744911

[29] Wheeler KM, Cárcamo-Oyarce G, Turner BS, Dellos-Nolan S, Co JY, Lehoux S, Cummings RD, Wozniak DJ, Ribbeck K. 2019. Mucin glycans attenuate the virulence of *Pseudomonas aeruginosa* in infection. Nat Microbiol 4:2146–2154. doi:10.1038/s41564-019-0581-8 31611643 PMC7157942

[30] Caldara M, Friedlander RS, Kavanaugh NL, Aizenberg J, Foster KR, Ribbeck K. 2012. Mucin biopolymers prevent bacterial aggregation by retaining cells in the free-swimming state. Curr Biol 22:2325–2330. doi:10.1016/j.cub.2012.10.028 23142047 PMC3703787

[31] Zhao K, Tseng BS, Beckerman B, Jin F, Gibiansky ML, Harrison JJ, Luijten E, Parsek MR, Wong GCL. 2013. Psl trails guide exploration and microcolony formation in *Pseudomonas aeruginosa* biofilms. Nature 497:388–391. doi:10.1038/nature12155 23657259 PMC4109411

[32] Ma L, Jackson KD, Landry RM, Parsek MR, Wozniak DJ. 2006. Analysis of *Pseudomonas aeruginosa* conditional psl variants reveals roles for the psl polysaccharide in adhesion and maintaining biofilm structure postattachment. J Bacteriol 188:8213–8221. doi:10.1128/JB.01202-06 16980452 PMC1698210

[33] Haley CL, Kruczek C, Qaisar U, Colmer-Hamood JA, Hamood AN. 2014. Mucin inhibits *Pseudomonas aeruginosa* biofilm formation by significantly enhancing twitching motility. Can J Microbiol 60:155–166. doi:10.1139/cjm-2013-0570 24588389 PMC4199588

[34] Kocevar-Nared J, Kristl J, Smid-Korbar J. 1997. Comparative rheological investigation of crude gastric mucin and natural gastric mucus. Biomaterials 18:677–681. doi:10.1016/s0142-9612(96)00180-9 9151999

[35] Huang Y, Mechref Y, Novotny MV. 2001. Microscale nonreductive release of O-linked glycans for subsequent analysis through MALDI mass spectrometry and capillary electrophoresis. Anal Chem 73:6063–6069. doi:10.1021/ac015534c 11791581

[36] Wolfgang MC, Kulasekara BR, Liang X, et al.. 2003. Conservation of genome content and virulence determinants among clinical and environmental isolates of Pseudomonas aeruginosa. Proc Natl Acad Sci U S A 100:8484–8489. doi:10.1073/pnas.0832438100 12815109 PMC166255

[37] Wolfgang MC, Kulasekara BR, Liang X, Boyd D, Wu K, Yang Q, Miyada CG, Lory S. 2003. Conservation of genome content and virulence determinants among clinical and environmental isolates of *Pseudomonas aeruginosa*. Proc Natl Acad Sci USA 100:8484–8489. doi:10.1073/pnas.0832438100 12815109 PMC166255

[38] Whitney JC, Colvin KM, Marmont LS, Robinson H, Parsek MR, Howell PL. 2012. Structure of the cytoplasmic region of PelD, a degenerate diguanylate cyclase receptor that regulates exopolysaccharide production in *Pseudomonas aeruginosa*. J Biol Chem 287:23582–23593. doi:10.1074/jbc.M112.375378 22605337 PMC3390633

[39] Jones CJ, Wozniak DJ. 2017. Psl Produced by Mucoid *Pseudomonas aeruginosa* Contributes to the Establishment of Biofilms and Immune Evasion. mBio 8:e00864-17. doi:10.1128/mBio.00864-17 28634241 PMC5478896

[40] Jones CJ, Wozniak DJ. 2017. Psl produced by mucoid *Pseudomonas aeruginosa* contributes to the establishment of biofilms and immune evasion. mBio 8:e00864-17. doi:10.1128/mBio.00864-17 28634241 PMC5478896

[41] Jacobs MA, Alwood A, Thaipisuttikul I, et al.. 2003. Comprehensive transposon mutant library of Pseudomonas aeruginosa. Proc Natl Acad Sci U S A 100:14339–14344. doi:10.1073/pnas.2036282100 14617778 PMC283593

[42] Kirisits MJ, Prost L, Starkey M, Parsek MR. 2005. Characterization of colony morphology variants isolated from Pseudomonas aeruginosa biofilms. Appl Environ Microbiol 71:4809–4821. doi:10.1128/AEM.71.8.4809-4821.2005 16085879 PMC1183349

[43] Zheng X, Gomez-Rivas EJ, Lamont SI, et al.. 2024. The surface interface and swimming motility influence surface-sensing responses in Pseudomonas aeruginosa. Proc Natl Acad Sci U S A 121. doi:10.1073/pnas.2411981121 PMC11441478 39284057

[44] Byrd MS, Sadovskaya I, Vinogradov E, Lu H, Sprinkle AB, Richardson SH, Ma L, Ralston B, Parsek MR, Anderson EM, et al.. 2009. Genetic and biochemical analyses of the *Pseudomonas aeruginosa* Psl exopolysaccharide reveal overlapping roles for polysaccharide synthesis enzymes in Psl and LPS production. Mol Microbiol 73:622–638. doi:10.1111/j.1365-2958.2009.06795.x 19659934 PMC4409829

[45] Irie Y, Starkey M, Edwards AN, Wozniak DJ, Romeo T, Parsek MR. 2010. *Pseudomonas aeruginosa* biofilm matrix polysaccharide Psl is regulated transcriptionally by RpoS and post-transcriptionally by RsmA. Mol Microbiol 78:158–172. doi:10.1111/j.1365-2958.2010.07320.x 20735777 PMC2984543

[46] Whitney JC, Whitfield GB, Marmont LS, Yip P, Neculai AM, Lobsanov YD, Robinson H, Ohman DE, Howell PL. 2015. Dimeric c-di-GMP is required for post-translational regulation of alginate production in *Pseudomonas aeruginosa*. J Biol Chem 290:12451–12462. doi:10.1074/jbc.M115.645051 25817996 PMC4432266

[47] Baraquet C, Harwood CS. 2016. FleQ DNA binding consensus sequence revealed by studies of FleQ-dependent regulation of biofilm gene expression in *Pseudomonas aeruginosa*. J Bacteriol 198:178–186. doi:10.1128/JB.00539-15 26483521 PMC4686206

[48] Hickman JW, Tifrea DF, Harwood CS. 2005. A chemosensory system that regulates biofilm formation through modulation of cyclic diguanylate levels. Proc Natl Acad Sci USA 102:14422–14427. doi:10.1073/pnas.0507170102 16186483 PMC1234902

[49] Nolan LM, McCaughey LC, Merjane J, Turnbull L, Whitchurch CB. 2020. ChpC controls twitching motility-mediated expansion of *Pseudomonas aeruginosa* biofilms in response to serum albumin, mucin and oligopeptides. Microbiology (Reading) 166:669–678. doi:10.1099/mic.0.000911 32478653 PMC7657506

[50] Schmidt WC, Lee CK, Zheng X, Chen JW, Fetah KL, Popoli JR, Choi YS, Young TD, Weiss PS, Kasko AM, et al.. 2025. *Pseudomonas aeruginosa* senses exopolysaccharide trails using type IV pili and adhesins during biofilm formation. Nat Microbiol 10:2511–2520. doi:10.1038/s41564-025-02087-4 40913087 PMC13267930

[51] Carey JN, Lamont S, Wozniak DJ, Dandekar AA, Parsek MR. 2024. Quorum sensing regulation of Psl polysaccharide production by *Pseudomonas aeruginosa*. J Bacteriol 206:e00312-24. doi:10.1128/jb.00312-24 39530727 PMC11656772

[52] Xin L, Zeng Y, Sheng S, Chea RA, Liu Q, Li HY, Yang L, Xu L, Chiam K-H, Liang Z-X. 2019. Regulation of flagellar motor switching by c-di-GMP phosphodiesterases in *Pseudomonas aeruginosa*. J Biol Chem 294:13789–13799. doi:10.1074/jbc.RA119.009009 31350333 PMC6746464

[53] Nicastro GG, Kaihami GH, Pulschen AA, Hernandez-Montelongo J, Boechat AL, de Oliveira Pereira T, Rosa CGT, Stefanello E, Colepicolo P, Bordi C, et al.. 2020. C-di-GMP-related phenotypes are modulated by the interaction between a diguanylate cyclase and a polar hub protein. Sci Rep 10:3077. doi:10.1038/s41598-020-59536-9 32080219 PMC7033161

[54] Ma L, Wang J, Wang S, Anderson EM, Lam JS, Parsek MR, Wozniak DJ. 2012. Synthesis of multiple *Pseudomonas aeruginosa* biofilm matrix exopolysaccharides is post-transcriptionally regulated. Environ Microbiol 14:1995–2005. doi:10.1111/j.1462-2920.2012.02753.x 22513190 PMC4446059

[55] Wang S, Yu S, Zhang Z, Wei Q, Yan L, Ai G, Liu H, Ma LZ. 2014. Coordination of swarming motility, biosurfactant synthesis, and biofilm matrix exopolysaccharide production in *Pseudomonas aeruginosa*. Appl Environ Microbiol 80:6724–6732. doi:10.1128/AEM.01237-14 25172852 PMC4249032

[56] Yu S, Wei Q, Zhao T, Guo Y, Ma LZ. 2016. A survival strategy for *Pseudomonas aeruginosa* that uses exopolysaccharides to sequester and store iron to stimulate Psl-dependent biofilm formation. Appl Environ Microbiol 82:6403–6413. doi:10.1128/AEM.01307-16 27565622 PMC5066357

[57] Murakami K, Ono T, Viducic D, Somiya Y, Kariyama R, Hori K, Amoh T, Hirota K, Kumon H, Parsek MR, et al.. 2017. Role of psl genes in antibiotic tolerance of adherent *Pseudomonas aeruginosa*. Antimicrob Agents Chemother 61:e02587-16. doi:10.1128/AAC.02587-16 28438927 PMC5487641

[58] Müller L, Murgia X, Siebenbürger L, Börger C, Schwarzkopf K, Sewald K, Häussler S, Braun A, Lehr C-M, Hittinger M, et al.. 2018. Human airway mucus alters susceptibility of *Pseudomonas aeruginosa* biofilms to tobramycin, but not colistin. J Antimicrob Chemother 73:2762–2769. doi:10.1093/jac/dky241 29982453

[59] Samad T, Co JY, Witten J, Ribbeck K. 2019. Mucus and mucin environments reduce the efficacy of polymyxin and fluoroquinolone antibiotics against *Pseudomonas aeruginosa*. ACS Biomater Sci Eng 5:1189–1194. doi:10.1021/acsbiomaterials.8b01054 33405639 PMC9267971

[60] Ershov D, Phan M-S, Pylvänäinen JW, Rigaud SU, Le Blanc L, Charles-Orszag A, Conway JRW, Laine RF, Roy NH, Bonazzi D, et al.. 2022. TrackMate 7: integrating state-of-the-art segmentation algorithms into tracking pipelines. Nat Methods 19:829–832. doi:10.1038/s41592-022-01507-1 35654950

[61] Parslow A, Cardona A, Bryson-Richardson RJ. 2014. Sample drift correction following 4D confocal time-lapse imaging. J Vis Exp 86:e51086. doi:10.3791/51086 PMC4166950 24747942

