# Supplementary material for: Mucin promotes Psl production and surface exploration in Pseudomonas aeruginosa
Source: mBio. 2026 Aug 13;17(9):e01445-26. doi: 10.1128/mbio.01445-26 (PMC13556220; doi:10.1128/mbio.01445-26)
Supplement: Supplementary file 1 [file mbio.01445-26-s0001.docx]

**Supplemental Figures**

**S1. Surface Associated α-Psl signal upon mucin addition**

**Fig S1: Mucin stimulates Psl trails:** PAO1 cells were exposed to a surface in the presence or absence of porcine gastric mucin at increasing concentrations for 1 h. Cells were then stained with α-Psl antibodies and imaged via widefield fluorescent microscopy. Total α-Psl signal was quantified per image in FIJI and made relative to the number of surface-associated cells.

**S2: α-Psl signal in 0.5% (w/v) CMC**

**Fig S2: α-Psl signal of surface associated cells in carboxymethylcellulose (CMC).** PAO1 cells were exposed to a surface in the presence or absence of 0.5% (w/v) CMC. Surface associated cells were then stained with α-Psl antibodies and imaged via widefield fluorescent microscopy. Total α-Psl signal was quantified per image in FIJI. Significance determined via Welch’s t test.

**S3: α-Psl signal over increasing glycan concentrations**

**Fig S3: α-Psl signal of surface associated Psl under increasing glycan concentrations.** PAO1 cells were exposed to a surface in the presence or absence of mucin-glycans at increasing concentrations for 1 h. Surface associated cells were then stained with α-Psl antibodies and imaged via widefield fluorescent microscopy. Total α-Psl signal was quantified per image in FIJI. Significance determined via Ordinary One-way ANOVA with Tukey’s multiple comparison test.

 **S4 : Δ*pilA* and Δ*pslBCD* twitching trajectories in VBMM and 0.5% (w/v) MUC**

0μm

>10μm

**Fig S4: Reconstructed trajectories of Δ*pslBCD* and Δ*pilA* in mucin.** Approximately 0.05OD PAO1, Δ*pslBCD*, or Δ*pilA* cells were added to VBMM +/- 0.5% (w/v) MUC in an Ibidi channel slide and the liquid surface interface was imaged via wide-field microscopy every 2.5 min for 60 minutes. Trajectories were reconstructed in Fiji using Trackmate. Scale bar = 10μm.

**S5. CFU counts of clinical isolates 1 h post-mucin incubation**

**Fig S5: CFU counts of clinical isolates 1 h post-mucin incubation.** Planktonic cells were incubated for 1 h in VBMM +/- 0.5% (w/v) MUC. Post-incubation, cells were plated for CFU counts. (N=3, n=3) Significance determined via 2-way ANOVA, * = p < 0.04.

**S3. Cell Free and Cell Associated Fractions + Ratio graph**

**Fig S6: Cell associated and Cell Free Psl Fractions in *P. aeruginosa* isolates**

**C.**

**A.**

**B.**

**Fig S6: α-Psl dot blots of clinical isolates in mucin.** Isolate cultures were incubated in VBMM +/- 0.5% (w/v) MUC for 1 h before separating free Psl vs cell-associated Psl fractions and dot blotting for Psl. **A)** Cell free Psl levels of varying isolates in VBMM or in MUC. **B)** Cell associated Psl levels of varying isolates in VBMM or in MUC. **C)** Ratio cell free vs cell associated Psl. n=3. Significance determines via 2way ANOVA, * = p < 0.05, ** = p < 0.005, *** = p < 0.0005, **** = p <0.0001

**S7. CFU quantification of *P. aeruginosa* in glycans**

**Fig S7: CFU quantification of *P. aeruginosa* in glycans.** PAO1 or PAO1 Δ*pslBCD* was incubated with 0.5% (w/v) porcine gastric mucin or 0.01% (w/v) mucin-glycans for 1 h before plating for CFU counts. n=3. Significance determined via 2way ANOVA, ns = non-significant

**S8. α-Psl dot blot of cell-free and cell associated Psl in mucin**

**Fig S8: α-Psl dot blot of cell-free and cell associated Psl in mucin.** PAO1 was incubated in increasing amounts of porcine gastric mucin (MUC) for 1 h before cell free and cell associated fractions were isolated and dot blotted with α-Psl. N=3, n=3.

**S9 Distance Traveled in PAO1 Δ*pslBCD* cells**

**Fig S9: Distance traveled in Psl-deficient cells in mucin.** PAO1 or Δ*pslBCD* was exposed to a surface in VBMM +/- 0.05% (w/v) MUC for 1 h while surface positions were tracked every 5 min via TrackMate extension in FIJI. n=3. Significance determined via One-way ANOVA, **** = p<0.0001.

References

**S10. CFU Counts for MIC Antibiotic Exposure in Mucin**

**A.**

**B.**

Δ*pslBCD*

PAO1

**Fig S10: Distance traveled in Psl-deficient cells in mucin. A)** PAO1 or Δ*pslBCD* was exposed to VBMM +/- 0.05% (w/v) MUC for 1 h before MIC H_2_O_2_ (2mM), Tobramycin (4μg/mL), or Ciprofloxacin (1μg/mL) was added for 6-8 hr before plating for viable CFU counts. n=3. Significance determined via Ordinary One-way ANOVA, with Tukey’s multiple comparison test. ** = p<0.01, * = p<0.05 **B)**  CFU counts of PAO1 or Δ*pslBCD* relative to the inoculum CFU at t=0 were plotted against increasing concentrations of H_2_O_2_, Tobramycin, or Ciprofloxacin. n=3. Significance determined via Ordinary 2-way ANOVA with with Šidák’s multiple comparison test.
